# Identification of single nucleotide polymorphism markers associated with resistance to bruchids (*Callosobruchus* spp.) in wild mungbean (*Vigna radiata* var. *sublobata*) and cultivated *V. radiata* through genotyping by sequencing and quantitative trait locus analysis

**DOI:** 10.1186/s12870-016-0847-8

**Published:** 2016-07-15

**Authors:** Roland Schafleitner, Shu-mei Huang, Shui-hui Chu, Jo-yi Yen, Chen-yu Lin, Miao-rong Yan, Bharath Krishnan, Mao-sen Liu, Hsiao-feng Lo, Chien-yu Chen, Long-fang O. Chen, Dung-chi Wu, Thu-Giang Thi Bui, Srinivasan Ramasamy, Chih-wei Tung, Ramakrishnan Nair

**Affiliations:** Biotechnology/Molecular Breeding, World Vegetable Center, 60 Yi Min Liao, Shanhua, Tainan 74151 Taiwan; Legume Breeding, World Vegetable Center, 60 Yi Min Liao, Shanhua, Tainan 74151 Taiwan; Information Technology, World Vegetable Center, 60 Yi Min Liao, Shanhua, Tainan 74151 Taiwan; Institute of Plant and Microbial Biology, Academia Sinica, No. 128, Sec. 2, Academia Road, Nankang, Taipei, 115 Taiwan; Horticulture and Landscape Architecture / Horticulture Section, Experimental Farm, College of Bio-Resources and Agriculture National Taiwan University, No. 1, Sec. 4, Roosevelt Road, Taipei, 106 Taiwan; Department of Bio-Industrial Mechatronics Engineering, National Taiwan University, No. 1, Sec. 4, Roosevelt Road, Taipei, 106 Taiwan; Division of Agricultural Biodiversity, Plant Resources Center, Plant Resources Center, Vietnam Academy of Agriculture Sciences, An Khanh, Hoai Duc, Ha Noi, Vietnam; Entomology, World Vegetable Center, 60 Yi Min Liao, Shanhua, Tainan 74151 Taiwan; Depertment of Agronomy, National Taiwan University, No. 1, Sec. 4, Roosevelt Road, Taipei, 106 Taiwan; Legume Breeding, World Vegetable Center South Asia, ICRISAT Campus, Patancheru, 502 324 Hyderabad, Telangana India

## Abstract

**Background:**

Bruchid beetles are an important storage pest of grain legumes. *Callosobruchus* sp. infect mungbean (*Vigna radiata*) at low levels in the field, multiply during grain storage and can destroy seed stocks in a few months. Resistance against bruchid beetles has been found in wild mungbean *V. radiata* var. *sublobata* TC1966 and in cultivated mungbean line V2802.

**Results:**

Bruchid resistance data were obtained from recombinant inbred line populations TC1966 (*V. radiata* var. *sublobata*) × NM92 (F_12_) and V2802 (*V. radiata*) × NM94 (F_7_). More than 6,000 single nucleotide polymorphic markers were generated through genotyping by sequencing (GBS) for each of these populations and were used to map bruchid resistance genes. One highly significant quantitative trait locus (QTL) associated with bruchid resistance was mapped to chromosome 5 on genetic maps of both populations, suggesting that TC1966 and V2802 contain the same resistance locus. Co-segregation of all markers associated with resistance indicated the presence of only one major resistance QTL on chromosome 5, while QTL analysis based on physical map positions of the markers suggested the presence of multiple QTLs on different chromosomes. The diagnostic capacity of the identified molecular markers located in the QTL to correctly predict resistance was up to 100 %.

**Conclusions:**

Molecular markers tightly linked to bruchid resistance loci of two different mungbean resistance sources were developed and validated. These markers are highly useful for developing resistant lines.

**Electronic supplementary material:**

The online version of this article (doi:10.1186/s12870-016-0847-8) contains supplementary material, which is available to authorized users.

## Background

Mungbean (*Vigna radiata*) is cultivated on about 6 million hectares, mainly in Asia. It is consumed as grains or as sprouts, the green pods are eaten as a vegetable, and it is processed into a variety of products such as noodles, sweets or drinks. Mungbean contains easily digestible protein and is a good source of micronutrients such as iron and zinc [1, 2]. High market demand commands relatively good farm gate prices for mungbean grain, making it a profitable rotation crop for Asian cereal production areas. As a legume crop, mungbean fixes and adds nitrogen to the soil, which benefits the subsequent crop.

Despite these benefits, expansion of the mungbean growing area is limited, mainly due to diseases and pests affecting the crop and reducing yield and profitability. One of the major insect pests of mungbean is bruchids. Beetles of the genera *Bruchus*, *Bruchidius*, *Callosobruchus*, *Acanthoscelides*, *Zabrotes* and *Caryedon* affect a range of legume grains including common bean (*Phaseolus vulgaris*), cowpea (*V. unguiculata*), mungbean (*V. radiata*), bambara groundnuts (*V. subterranea*), chickpea (*Cicer arietinum*), pigeon pea (*Cajanus cajan*) and other grain legumes [3]. The major mungbean infecting bruchid species in Asia are *Callosobruchus chinensis* and *C. maculatus* [4]. These pests first infect the grain in the field, at low levels. During grain storage, they develop from egg to pupa in a single seed, the larva being the most destructive stage. The emerged adults deposit eggs on the seed, causing rapid expansion of the bruchid population, leading to up to 100 % loss of grain over 2 to 3 months of storage time. The grain stored for sale is destroyed, and the farmers also lose seed for the next season’s planting. To avoid storage losses, farmers tend to sell the grain immediately after harvest when the price is lowest, reducing their profit. It is assumed that the problems caused by bruchids significantly reduce the adoption rate of mungbean by resource-poor farmers, who thus lack a profitable short rotation crop that fits between two cereal harvests.

Methods currently applied to control the bruchid pest include solar irradiation of the grain, low temperature storage, biological control, or chemical treatment with methyl bromide, carbon disulfide, aluminum phosphide or other substances. Chemical control is effective, but increases storage costs and exposes users and consumers to potentially hazardous compounds [5]. Bruchids may develop resistance against the chemicals over time. Host resistance to bruchids would be the most sustainable way to control the pest. Bruchid resistance in legumes relies on morphological barriers preventing colonization of the seed by bruchid larvae, or on secondary metabolites and other possibly toxic compounds interfering with bruchid growth, development or reproduction [6]. Bruchid resistance factors have been isolated from bean and chickpea seeds [7–9]. In rice bean (*V. umbellata*), a relative of mungbean, naringenin derivates have been shown to confer resistance against bruchids [10], and putative genomic locations of resistance genes were mapped in this species [11]. Complete bruchid resistance in mungbean has been found in the wild relative *V. radiata* var. *sublobata* TC1966 [4]. One major and two minor bruchid resistance genes have been mapped in this line [12]. Recently, [13] confirmed the presence of resistance genes against bruchids on chromosome 5 of TC1966. In mungbean line VC6089A, which was bred by using TC1966 as a resistance source, a protein putatively having polysaccharide hydrolase activity termed VrD1 was isolated, which inhibited the development of *C. maculatus* into adults when used in artificial seeds [14]. A 4-week feeding study on mice comparing a commercial mungbean line with an isogenic line containing the bruchid resistance gene from TC1966 showed no negative impact on growth or any pathological effect of the *V. radiata var. sublobata* TC1966 bruchid resistance gene product on the animals [15]. However, bruchid resistance in *V. radiata* var. *sublobata* seems to be linked with undesirable seed properties, such as small and hard seed [16, 17]. Breaking the linkage between bruchid resistance and the small and hard seed phenotype has been demonstrated, but it was found that bruchid resistance in TC1966 is linked in the repulsion phase to an important resistance gene against *Mungbean yellow mosaic virus* derived from line NM92 [12]. For all these reasons, breeders are reluctant to use TC1966 as a bruchid resistance source. Alternative resistance sources would increase the options available for breeding bruchid resistant mungbean.

Screening of cultivated mungbean germplasm at the World Vegetable Center for complete resistance to *C. chinensis* and *C. maculatus* yielded two resistant accessions, V2709 and V2802 [18]. Wild black gram (*V. mungo* var. *silvestris*) VM2164 is another potential donor for bruchid resistance genes [19, 20]. V2709 has been used in Korea to breed the bruchid-resistant variety Jangan and two quantitative trait loci (QTL) conferring resistance were identified in this line [21]. The same resistance source was also used in China to create bruchid-resistant lines Zhonglv 3, Zhonglv 4 and Zhonglv 6 [22]. Lines carrying the V2709 resistance gene were suggested to be safe for human consumption based on an animal oral toxicity study [22]. Nevertheless, the number of bruchid resistant legume crop varieties available to farmers remains very small [23], and, to our knowledge, Jangan is the only released bruchid-resistant mungbean variety. More information on the biochemical basis of bruchid resistance and feeding studies assessing the safety of alternative resistance sources are required to guarantee safety of bruchid-resistant mungbean for human nutrition.

Breeding of bruchid-resistant legumes is a laborious task. Reconstructing the elite line phenotype after resistance introgression may require several generations of backcrossing due to linkage drag, while resistance screening at each back-cross generation through bioassays is costly and error-prone (reviewed by [23]). Molecular markers tightly associated with resistance would improve selection efficiency, drastically reduce the number of required resistance tests, and greatly lower the selection costs. Markers linked to bruchid resistance of TC1966 and V2709 have been identified by [12, 13, 21]. For the alternative resistance source V2802, no information on the chromosomal location of the resistance gene(s) and no markers associated with these loci were available. Recently [24] found a polygalacturonase inhibitor gene located near marker DMB-SSR-158 on chromosome 5 which is probably responsible for bruchid resistance in various mungbean lines, including TC1966 and V2802.

The genotyping by sequencing (GBS) technology is highly efficient for producing large numbers of single nucleotide polymorphism (SNP) markers for virtually any organism [25]. Together with the available whole genome information of mungbean [26], this technology greatly facilitated quantitative trait locus (QTL) analyses to identify markers associated with a trait of interest such as bruchid resistance. The present study applied GBS on populations derived from crosses of bruchid resistant wild mungbean TC1966 and cultivated mungbean V2802 with bruchid susceptible lines NM92 and NM94 to identify and compare resistance loci between the two different resistance sources.

## Results

### Bruchid resistance segregation in the experimental populations

**Fig. 1:**
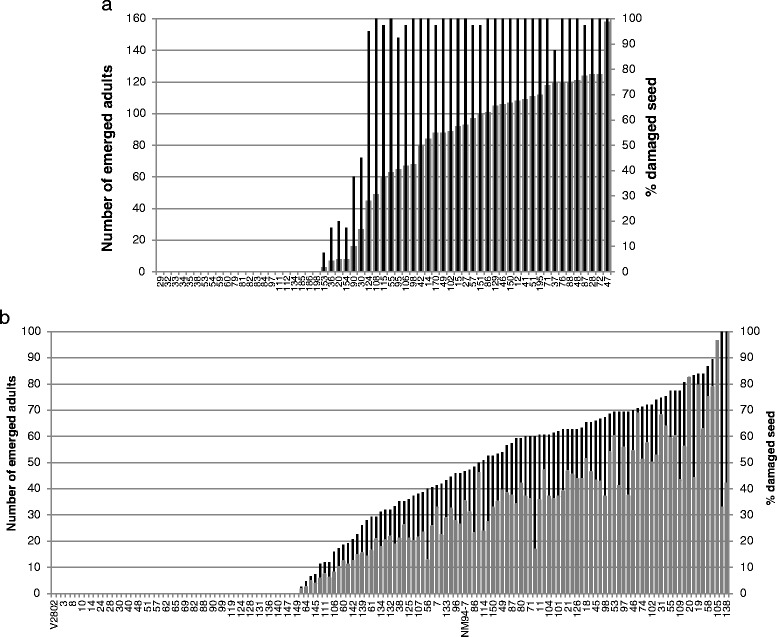
Response to bruchids of F_12_ families of TC1966 × NM92 (**a**) and of F_7_ families of V2802 × NM94 (**b**). Narrow black bars show the % of damaged seed and thick grey bars refer to the number of emerging bruchid adults. The numbers along the x-axis designate the family numbers

Sixty-one F_12_ families of TC1966 × NM92 were tested for bruchid resistance. Twenty-two families were 100 % resistant, showing no damaged seed and no emerging beetles, while 33 families had more than 90 % damaged seeds and between 40 and 98 beetles emerged from the seed batches during resistance testing. Six families with intermediate phenotypes had between 7.5 and 45 % damaged seed and between 3 and 45 emerging beetles (Fig. 1a). The segregation pattern suggested the action of a major resistance gene supported by genes modulating resistance, explaining the presence of intermediate resistant phenotypes in homozygous recombinant inbred line (RIL) families.

In V2802 × NM94, the number of completely resistant RIL families increased from the F_3_ to the F_7_ generation, from 13 to 52. Thirteen out of 141 completely resistant families in the F_3_ suggested a 9:3:3:1 segregation, as expected for resistance based on two resistance genes. Generation advancement by single seed descent led to increased homozygous plants, raising the number of completely resistant and susceptible families in the subsequent generations. From 141 F_7_ families, 52 were completely resistant with 0 % seed damage and no emerging beetles, 64 had more than 40 % damaged seed, and 25 families showed intermediate damage to 3 to 39 % seed (Fig. 1b). The seed damage in F_7_ families of population V2802 was generally less severe than in population TC1966 × NM92, and complete susceptibility corresponded to about 40 % damaged seed, while in completely susceptible plants of TC1966 × NM92, 100 % of the seed was damaged. This result indicates that either V2802 carries stronger resistance genes than TC1966, or NM94 contributed to resistance of the progenies.

### Genotyping by sequencing

For population TC1966 × NM92, 56,154,121 sequencing reads, each 101 bp long, were obtained and 48,105,477 reads with the barcode followed by the restriction site remnant and no ambiguous base in the first 64 bp were mapped to 258,151 unique sites of the mungbean reference genome [26]. In total, 32,856 SNPs were obtained and 9,282 SNPs were scored in at least two-thirds of the RILs. 7,460 of the SNPs were aligned to the 11 chromosomes of mungbean, and 1,822 aligned to scaffold sequences that could not yet be integrated into chromosomes of the reference genome. For cross V2802 × NM94 (F_7_) 437,644,283 reads were obtained from 141 F_7_ plants and 2 parental lines, and 376,822,250 reads containing full barcode and restriction remnant sites were aligned to 934,484 unique sites of the mungbean reference genome [26]. 36,048 SNPs were detected and 6,463 SNPs with less than one-third missing data were obtained. Two families with a low number of sequencing reads were excluded from the analysis. The SNPs of both population that could be mapped to the 11 chromosomes of the reference genome are listed in Additional file 1: Table S1.

### QTL analysis

Based on the physical position of all SNP markers with less than 30 % missing data, inclusive composite interval mapping in population TC1966 × NM92 pinpointed a significant QTL interval for reduced seed damage on chromosome 5, ranging from position 5,178,332 to 5,179,402 (logarithm of odds [LOD]: 36.4, explaining 43.3 % of the variation and an additive effect of −31.2 % seed damage). This QTL co-localized with an interval highly associated with reduced number of emerging adults (LOD: 28.4, 80.9 % of the variation explained and an additive effect of −18.5 adults). Also in V2802 × NM94, inclusive composite interval mapping resulted in a single significant QTL location for reduced seed damage and number of emerging bruchids on chromosome 5; however, the physical map positions of the markers flanking the QTL were located at positions 5,877,096 and 5,953,917. The LOD for the seed damage and emerging bruchid number QTLs were 41.2 and 52.9 and the % variation was 74.8 and 82.9 %, respectively; the additive effect was −27.0 % seed damage and −8.41 emerging bruchid beetles. In addition, a second QTL physically mapped to chromosome 4 between positions 15,343,475 and 15,429,977 with an LOD of 39.9 and 27.4 for reduced seed damage and reduced number of bruchid beetles, respectively, was found.

**Fig. 2:**
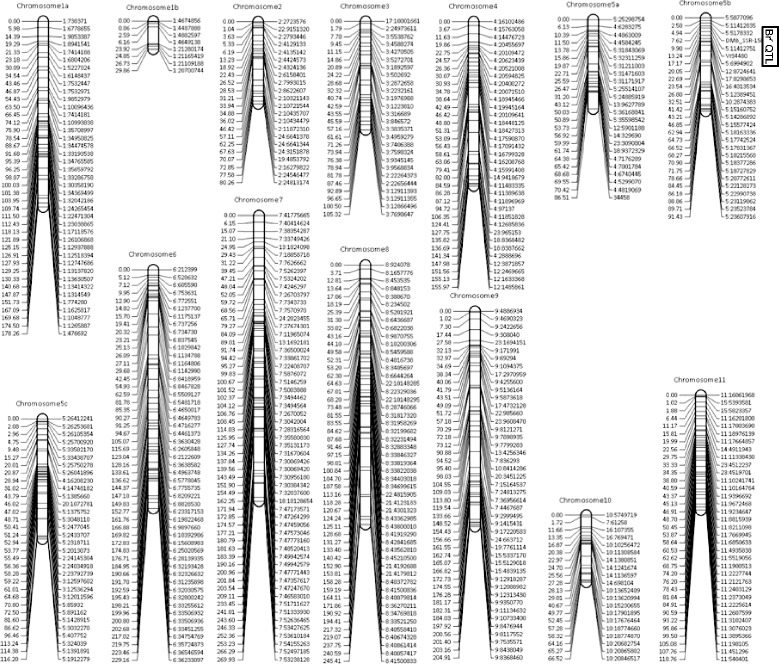
Genetic map of TC1966 × NM92

QTL mapping was repeated on genetic maps generated for TC1966 × NM92 and V2802 × NM94 to include markers mapping to scaffolds that were not yet included in one of the 11 chromosomes, and to account for possible differences in marker order between the experimental population and the sequenced mungbean line VC1973 [26]. The 9,289 markers for TC1966 × NM92 were grouped into 476 bins spanning a map extending 1,978 centimorgan (cM) along 14 linkage groups, where chromosome 1 was split into two and chromosome 5 into three linkage groups (Fig. 2). The marker order of the genetic map differed strongly from the order according to the physical map, probably due to the small population size, but possibly also due to rearrangements in the TC1966 and NM92 genomes relative to the sequenced line VC1973. Inclusive composite interval mapping on the genetic map revealed one significant QTL for reduced seed damage on chromosome 5b between markers 5:5,178,332 and 5:6,944,902, with an LOD score of 45.8, explaining 97.1 % of the variation of % and contributing an additive effect of −46.8 %. A QTL for reduced number of bruchid adults was located at the same position, with an LOD of 32.0 explaining 91.7 % of the trait variation and an additive effect of −20.7 emerging adult bruchids. The marker bins flanking and located in the QTL interval contained, in addition to 81 markers physically mapped to chromosome 5, 87 markers physically mapped to positions 10,421,576 to 12,504,219 of chromosome 3 and 14 markers physically mapped to positions 15,135,409 to 15,429,977 of chromosome 4 of the reference genome. Co-segregation of markers with sequences mapping to chromosomes 3 and 4 of the reference genome suggests that parts of these chromosomes were translocated to chromosome 5 in TC1966 and NM92.

**Fig. 3:**
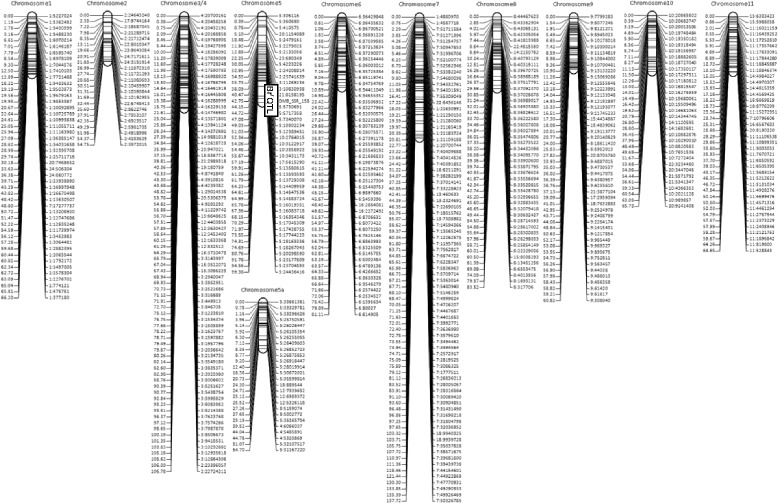
Genetic map of V2802 × NM94

In total, 6,463 markers for V2802 × NM94 were grouped into 510 bins and resulted in a genetic map spanning 875 cM along 11 linkage groups corresponding to chromosomes 1, 2 and 5 to 11 (Fig. 3). Chromosomes 3 and 4 were merged into one linkage group, while chromosome 5 was split into two linkage groups. Several markers physically mapping to chromosome 2, 3 and 4 of the reference genome mapped to chromosome 5. In general, the marker order along the genetic map was highly divergent from expected order of the markers according to their physical map position on the reference sequence. One QTL for both seed damage and number of emerging bruchids was located on chromosome 5 between markers 3:10,830,930 and 5:5,730,691 with an LOD of 41.3 and 53.1, respectively, explaining 74.8 and 82.9 % of the variation and an additive effect of −27.0 % seed damage and −8.1 emerging bruchids. Marker 3:10,830,930 was physically mapped to chromosome 3 but was tightly linked to markers on chromosome 5. The marker bins located at this QTL contained 51 markers physically mapped to chromosome 5, 30 to chromosome 4 (position 15,135,409 to 15,572,752) and 7 to chromosome 3 (10,421,576 to 10,579,209) of the reference genome sequence.

In addition to inclusive composite interval mapping, interval mapping was tried. It yielded, in addition to the QTL on chromosome 5, putative QTLs on chromosomes 1, 7 and 10 in TC1966 × NM92, and QTLs on chromosomes 2 and 10 in V2802 × NM94 (Additional file 2: Table S2).

#### QTL verification

Two steps of QTL data validation were performed. First, the GBS SNP data along the bruchid resistance QTLs were verified in the experimental populations using polymerase chain reaction (PCR)-based markers. Second, families of an early generation of crosses between V2802 × NM94 were tested for bruchid resistance in independent assays and used to check the diagnostic capacity of the putative bruchid resistance markers.

Inclusive composite interval mapping on genetic maps yielded a strong QTL for bruchid resistance on chromosome 5 of both populations, while QTL analysis on physical maps gave an additional QTL on chromosome 4 for V2802 × NM94. In addition, the chromosome 5 QTLs detected on genetic maps of both populations contained also markers that physically mapped to chromosomes 3 and 4. Therefore, markers from chromosomes 3, 4 and 5 were chosen for validation. Markers in or flanking the QTL intervals were converted to CAPS or dCAPS markers and genotyped in the mapping population. A SNP marker physically mapping to position 10,830,930 of chromosome 3 and delimiting the chromosome 5 QTL on the genetic map of V2802 × NM94 could not be converted to a PCR-based marker. Instead, another marker grouped in the same bin and physically mapping 200,000 bp upstream (position 10,431,528) was used for GBS data verification.

**Fig. 4:**
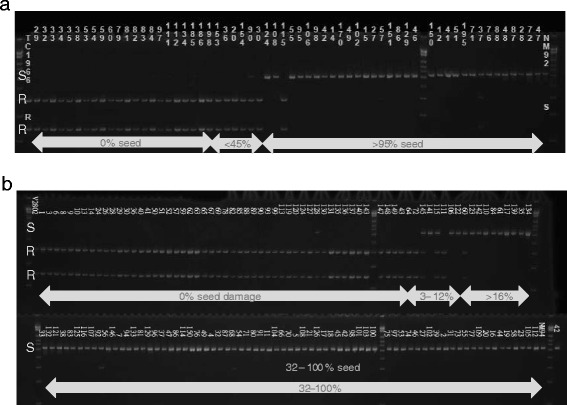
Genotypes of marker CAPS12 detecting the bruchid resistance allele in populations TC1966 × NM92 (**a**) and V2802 × NM94 (**b**) ordered by resistance in terms of % seed damage. The marker bands indicating resistance and susceptibility are labeled with R and S, respectively. In population V2802 × NM94 family 128 has a low proportion of the susceptible allele and family 92 has a low proportion of the resistant allele. In all other families marker CAPS12 correctly predicts resistance or susceptibility

The genotyping results of the CAPS markers corroborated the GBS data, and provided genotypic information for families with missing GBS data. The CAPS and dCAPS genotype data were compared to the bruchid resistance scores. Table 1 shows the rate of correct prediction of the bruchid resistance phenotype in the mapping populations. In both populations CAPS markers physically mapping to chromosome 3, 4 and 5 were highly diagnostic and predicted resistance and susceptibility correctly in both populations. The markers having the highest co-segregation rate (>98 %) with resistance in population TC1966 × NM92 were physically mapped to chromosome 3 at position 10,431,528 bp, chromosome 4 at position 15,255,162 bp, and to chromosome 5 from position 5,178,332 to 5,179,402 bp, and again on chromosome 5 from 5,953,917 to 7,551,254 bp. In V2802 × NM94, markers physically mapped at 5,622,070, 5,662,479, 5,953,917 and 5,974,663 were 100 % co-segregating with resistance phenotype. The marker genotypes for CAPS12 depicting the diagnostic capacity of this marker in both populations is shown in Fig. 4. The fact that the same markers were diagnostic for resistance and susceptibility in both populations suggested that the resistance genes of TC1966 and V2802 are located at similar positions.

**Table 1:** Diagnostic capacity of PCR-based markers located in the bruchid resistance QTLs of TC1966 x NM92 and V2802 x NM94

Marker	Chromosome	Position (bp.)	Correct prediction of resistance/susceptibility (% of RILs)	
			TC1966 × NM92 (F_12_)	V2802 × NM94 (F_7_)
dCAPS2	3	10,431,528	98.4	98.5
dCAPS3	4	15,255,162	98.4	98.5
CAPS1	5	5,178,332	98.4	98.5
CAPS2	5	5,179,402	98.4	99.3
dCAPS1	5	5,454,538	-	98
CAPS3	5	5,622,070	93.4	100
CAPS4	5	5,662,479	93.4	100
CAPS6	5	5,730,691	93.4	99.28
CAPS12	5	5,953,917	98.4	100
CAPS13	5	5,974,663	-	100
CAPS14	5	6,066,948	98.4	99.28
CAPS8	5	6,992,170	98.4	98.5
CAPS9	5	7,212,649	98.4	97.8
CAPS11	5	7,551,254	98.4	97.8

Subsequently, the markers tightly associated with bruchid resistance were tested in 86 F_3_ families of V2802 × NM94. Markers dCAPS2, dCAPS3, CAPS1 and CAPS12 correctly predicted resistance; all resistant families were homozygote for the resistant allele, while susceptible plants were homozygote for the susceptible allele (Table 2). As already observed in F_7_ families, the four markers, although physically mapped to different chromosomes, co-segregated in the F_3_ families at a high proportion (96.5 %), indicating genetic linkage between the markers.

**Table 2:** Validation of markers dCAPS2, dCAPS3, CAPS1 and CAPS12 in the F3 generation of V2802 × NM94

Family number	% damage	dCAPS2	dCAPS3	CAPS1	CAPS12	Family number	% damage	dCAPS2	dCAPS3	CAPS1	CAPS12
9	0	RR	RR	RR	RR	59	50.0	H	H	H	H
14	0	RR	RR	RR	RR	46	53.3	H	H	H	H
24	0	RR	RR	RR	RR	**56**	56.7	H	H	H	H
30	0	RR	RR	RR	RR	27	63.3	H	H	H	H
36	0	RR	RR	RR	RR	18	26.7	SS	SS	SS	SS
50	0	RR	RR	RR	RR	16	33.3	SS	SS	SS	SS
**52**	0	**SS**	**H**	**RR**	**RR**	84	36.7	SS	SS	SS	SS
69	0	RR	RR	RR	RR	55	40.0	SS	SS	SS	SS
89	0	RR	RR	RR	RR	33	48.2	SS	SS	SS	SS
93	0	RR	RR	RR	RR	47	50.0	SS	SS	SS	SS
72	0	RR	RR	RR	RR	66	50.0	SS	SS	SS	SS
51	6.7	RR	RR	RR	RR	42	53.3	SS	SS	SS	SS
1	10.00	RR	RR	RR	RR	96	60.0	SS	SS	SS	SS
15	16.7	RR	RR	RR	RR	71	60.0	SS	SS	SS	SS
29	20.0	RR	RR	RR	RR	100	60.0	SS	SS	SS	SS
65	23.3	RR	RR	RR	RR	37	65.2	SS	SS	SS	SS
82	23.3	RR	RR	RR	RR	86	70.0	SS	SS	SS	SS
90	23.3	RR	RR	RR	RR	70	70.0	SS	SS	SS	SS
43	23.3	RR	RR	RR	RR	91	73.3	SS	SS	SS	SS
6	33.3	RR	RR	RR	RR	98	73.3	SS	SS	SS	SS
13	36.7	RR	RR	RR	RR	102	76.6	SS	SS	SS	SS
48	36.7	RR	RR	RR	RR	54	76.7	SS	SS	SS	SS
**67**	**40.0**	**RR**	**H**	**H**	**RR**	83	80.0	SS	SS	SS	SS
10	13.3	H	H	H	H	7	80.0	SS	SS	SS	SS
41	23.3	H	H	H	H	94	80.0	SS	SS	SS	SS
85	26.7	H	H	H	H	4	80.0	SS	SS	SS	SS
40	30.0	H	H	H	H	32	82.9	SS	SS	SS	SS
57	40.0	H	H	H	H	76	83.3	SS	SS	SS	SS
99	40.0	H	H	H	H	31	83.3	SS	SS	SS	SS
26	46.7	H	H	H	H	20	86.7	SS	SS	SS	SS
63	53.3	H	H	H	H	61	86.7	SS	SS	SS	SS
62	70.0	H	H	H	H	58	86.7	SS	SS	SS	SS
60	50.0	H	H	H	H	53	90.0	SS	SS	SS	SS
**81**	**90.0**	**RR**	**RR**	**RR**	**SS**	97	90.0	SS	SS	SS	SS
75	13.3	H	H	H	H	11	93.3	SS	SS	SS	SS
77	13.3	H	H	SS	SS	5	93.3	SS	SS	SS	SS
35	16.7	H	H	H	H	21	93.3	SS	SS	SS	SS
23	16.7	H	H	H	H	17	93.3	SS	SS	SS	SS
49	23.3	H	H	H	H	19	93.3	SS	SS	SS	SS
74	23.3	H	H	H	H	73	93.3	SS	SS	SS	SS
101	36.7	H	H	H	H	38	96.7	SS	SS	SS	SS
44	40.0	H	H	H	H	80	100.0	SS	SS	SS	SS
39	43.3	H	H	H	H	34	100.0	SS	SS	SS	SS

RR, SS: homozygote resistance and susceptibility allele, respectively. H: heterozygote. %damage: Percent of damaged seed in bruchid resistance assays. Loci where the 4 tested markers did not co-segregate are printed in bold

In addition to QTLs obtained from inclusive composite interval mapping, also resistance QTL loci detected by interval mapping were verified. Tetra markers were designed for four putative QTLs located on chromosomes 1, 2, 7 and 10. In TC1966 × NM92 (F_12_) the correct prediction rate of tetra marker 1, 3 and 4 assessing the SNP genotype in putative QTLs on chromosomes 1, 7 and 10 amounted to 97, 70 and 80 % respectively. Tetra marker 2 testing a SNP on chromosome 2 and Tetra marker 4 assessing a SNP on chromosome 10 predicted resistance and susceptibility correctly in 90 and 85 %, respectively, in 130 families of V2802 × NM94 (F_7_). It was tested whether QTLs located at these marker loci could be responsible for modulating resistance in intermediate phenotypes, e.g. by conferring susceptibility alleles in families that carry the resistance allele at the chromosome 5 QTL or vice versa. In both populations the markers associated with putative QTLs on chromosomes 1, 2, 7 and 10 co-segregated with the genotypes of markers linked to the chromosome 5 QTL (Additional file 5: Figure S1). This result suggested that these putative QTLs did not play a role in modulating resistance in families with intermediate phenotypes. Markers previously described to be associated with bruchid resistance showed a segregation pattern similar to other chromosome 5 QTL-linked markers and failed to explain the intermediate phenotypes (Additional file 3: Table S3).

**Fig. 5:**
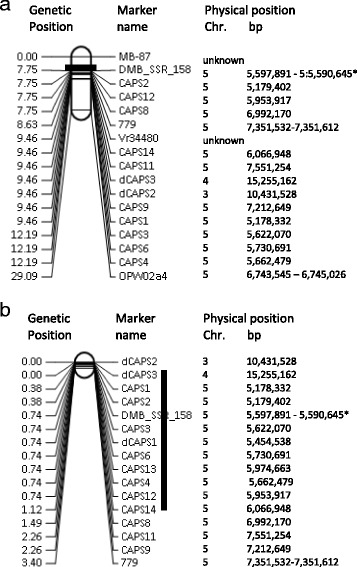
Order of markers putatively associated with bruchid resistance in TC1966 × NM92 (**a**) and V2802 × NM94 (**b**). The QTL intervals are indicated by black bars. *) The primers for DMB-SSR-158 map 7,000 bp apart on the VC1973 reference genome sequence

The order of the CAPS and dCAPS markers of our study and of markers previously found being associated with bruchid resistance was assessed on genetic maps (Fig. 5). In TC1966, 15 bruchid-resistant markers spanned 4.44 cM. The order of the markers differed between the genetic and the physical map. In addition, markers physically mapped to chromosomes 3 and 4 were strongly linked to markers mapped to chromosome 5. In V2802 × NM94 16 markers spanned 3.4 cM and the marker order between genetic and physical map was less different than for TC1966 × NM92, but here as well markers of chromosomes 3 and 4 clustered with markers on chromosome 5. Marker DMB-SSR-158 previously found associated with bruchid resistance clustered with diagnostic markers in both populations. In contrast, markers Mb-87 and OPW02a4 described being associated with bruchid resistance in V2709 [21] and TC1966 [27] mapped 7.75 and 16.09 cM away from the nearest chromosome 5 QTL-related marker. Three gene-based markers recently found associated with resistance in TC1966 × NM92 [13] were also tested in V2802 x NM94 (Additional file 3: Table S3). Markers 779 and Vr34480 were co-segregating with chromosome 5 QTL-related markers and marker 34458 was located in gene Vr5g03830.1 [13], which was positioned in the chromosome 5 QTL interval. Markers Vr34480 and 34458 were dominant. The resistance phenotype prediction accuracy in V2802 × NM94 was 99 % for 34458, 96.5 % for Vr34480, and 94 % for 779. Inclusive composite interval mapping using the CAPS markers suggested the strongest association with bruchid resistance at position 7.0 cM in TC1966 × NM92 and at position 1 cM of V2802 × NM94, between markers dCAPS3 and CAPS14.

Genetic mapping suggested that markers physically mapped to chromosomes 3 and 4 and associated with bruchid resistance map in fact to chromosome 5. To assess whether all markers associated with bruchid resistance by inclusive composite interval mapping indeed map to chromosome 5, the primer and amplification product sequences of markers dCAPS 2 and dCAPS 3 were mapped to available mungbean sequences. Both primer and fragment sequences of dCAPS2 and dCAPS3 were unambiguously mapped to chromosomes 3 and 4 of reference sequence VC1973, respectively. When using the sequence scaffolds of recombinant inbred line RIL59 derived from TC1966 × NM92 as a reference [13], both markers mapped to scaffold 35, which was attributed to chromosome 3 of RIL59. This result indicates that 1) there was a chromosomal rearrangement in TC1966 × NM92 in comparison to the reference sequence concerning at least sections of chromosomes 3 and 4, and 2) there should be a second bruchid resistance locus on chromosome 3 on population TC1966 × NM92 pinpointed by markers dCAPS2 and 3.

The mungbean reference sequence was inspected for the gene content of the QTL interval on chromosome 5. Despite the probable rearrangement of sections of chromosome 5 relative to the reference sequence of VC1973, the gene content between positions 5,178,332 and 6,066,948 of chromosome 5 could indicate a possible bruchid resistance gene. Through GBS, 7 SNPs were found in the region of gene Vradi05g03780.1, and 4 of them predicted an amino acid sequence changes in this gene. Further SNPs potentially leading to amino acid sequence changes in proteins were found in Vradi05g03980.1 and Vradi05g04130.1 (Additional file 4: Table S4). Interestingly, both V2802 and TC1966 exhibited sequence variations in the probable LRR receptor-like serine/threonine-protein kinase Vradi05g04130.1 predicting differences in the amino acid sequence compared to bruchid susceptible lines NM92 and NM94 (Additional file 4: Table S4). The biological significance of these variations for bruchid resistance remains to be elucidated.

## Discussion

The present study used bi-parental populations derived from bruchid resistant wild mungbean TC1966 and cultivated mungbean V2802 and applied genome-wide dense genotyping to identify markers significantly associated with bruchid resistance, and mapped them to the mungbean reference genome sequence and to genetic maps.

Strong segregation of resistance was found in both experimental populations TC1966 × NM92 and V2802 × NM94, ranging from 100 % resistance to 100 % susceptibility. QTL analysis using inclusive composite interval mapping on genetic maps revealed one highly significant locus associated with bruchid resistance in both populations. Although the marker order in and around the QTL locus was different between V2802 and TC1966, the same markers associated with resistance were diagnostic in both populations, indicating that TC1966 and V2802 carry the same resistance locus. When resistance is based on a single gene, it should segregate in a 1:3 ratio in the F_2_ generation and intermediate phenotypes should be absent in highly homozygote F_7_ and F_12_ families. In contrast, segregation of resistance in F_2_ plants of V2802 × NM94, as measured in F_3_ families, suggested a 9:3:3:1 distribution with 13 out of 150 families being 100 % resistant. Furthermore, families with intermediate resistance were present in both recombinant inbred populations, strongly suggesting the action of at least two resistance genes. TC1966 × NM92 families carrying the resistance allele for chromosome 5 showed higher seed damage than V2802 × NM94 families, suggesting a contribution of NM94 to resistance. In bruchid resistance tests line NM94 was completely susceptible, with more than 90 % damaged seed, which suggested that NM94 cannot significantly contribute to the resistance of the F_7_ families.

The markers associated with the QTL identified on genetic maps of both populations also contained markers physically mapping to other chromosomes of the VC1973 reference sequence. Two markers associated with resistance, dCAPS2 and dCAPS3, which mapped to chromosome 3 and 4 of the reference sequence VC1973, were both mapped to one scaffold of the sequenced TC1966 × NM92 recombinant inbred line RIL59 [13]. These mapping data suggested the presence of two resistance loci. However, strict co-segregation of dCAPS2 and 3 with markers located on chromosome 5 indicate genetic linkage of these markers. On the other hand, co-segregation of markers located on different chromosomes could also have biological reasons, such as the presence of segregation distorter genes nearby [28]. But a possible QTL on chromosome 3 that co-segregates with chromosome 5 cannot explain the presence of intermediate phenotypes. Both interval and inclusive composite interval mapping failed to identify modifier genes required to explain the presence of intermediate phenotypes in highly homozygote recombinant inbred lines that were produced by single seed descent. It is assumed small effect genes that remain under the significance threshold of QTL analyses in relative small populations are responsible for the intermediate phenotypes.

Bruchid resistance in TC1966 has been mapped previously. It has been found associated with RAPD marker fragment OPW02a4 [27], which mapped to position 6,743,539 to 6,745,030 of chromosome 5 of the mungbean reference genome. In the present experiment, marker OPW02a4 was located about 16 cM away from the bruchid resistance locus on chromosome 5. The diagnostic rate of the marker in TC1966 × NM92 F_12_ families was 87 %. The marker mainly failed to correctly detect 100 % resistant and susceptible genotypes and thus seemed to modulate resistance in intermediate phenotypes. Chen et al. [12] reported one major and two minor QTLs. The major QTL was linked to microsatellite marker DMB-SSR-158. The primers of this marker map at a distance of more than 7,000 bp from each other on the VC1973 reference genome sequence, but yielded PCR fragments between 300 and 400 bp in size, indicating again rearrangement along chromosome 5 in the lines used by this study compared to VC1973. DMB-SSR-158 mapped around position 5,598,000 bp of chromosome 5, and on genetic maps of TC1966 × NM92 and V2802 × NM94 the marker was located either in or very near to the chromosome 5 QTL interval. Recently, a polygalacturonase inhibitor gene located at this position was suggested to be responsible for bruchid resistance in mungbean [24]. The minor bruchid resistance QTLs published in [12] could not be verified, as these QTLs were delimited by an amplified fragment length polymorphism (AFLP) markers and no sequence information for converting these markers to PCR-based markers was available.

Hong et al. [21] mapped bruchid resistance of a different mungbean line (V2709) to intervals defined by marker pairs MB-87 – COPU11 and RP –COPU06. Alignment of marker MB-87 to the reference sequence was ambiguous, probably because the markers were derived from a different *Vigna* species and partial sequence similarity with *V. radiata* may have led to amplification of different fragments than suggested by sequence similarity analysis with the mungbean reference genome. MB-87 was polymorphic in population TC1966 × NM92, and mapped 7.5 cM away from the bruchid resistance locus. It was associated with bruchid resistance in 88.5 % of 61 tested families. Marker RP was polymorphic in the population, but the low quality of the obtained bands did not allow reliable scoring for this marker. Liu et al. [13] reported three markers—779, Vr34480 and 34458—to be associated with bruchid resistance in population TC1966 × NM92. Two of these markers were dominant in population V2802 × NM94 and all three markers were highly diagnostic for bruchid resistance in V2802 × NM94.

The marker order in the two mapping populations was strongly different from the order suggested by the mungbean whole genome sequence of VC1973. Kang et al. [26] demonstrated some degree of variation in scaffold alignments between VC1973 and TC1966. Liu et al. [13] showed that there are large variations in genome size of different mungbean lines and demonstrated chromosomal rearrangements in TC1966 compared to the reference sequence of VC1973, especially on chromosome 5. Nevertheless, additional ambiguity in genetic mapping of markers in TC1966 × NM92 may be due to the small population size.

Markers associated with resistance have been made available. Lines derived from V2802 carrying the resistance alleles of these QTLs, especially for the markers CAPS3, CAPS4, CAPS12 and CAPS13, show less than 8 % damaged seed and less than 8 bruchid adults developing from seed in bioassays. These markers are currently used in the World Vegetable Center breeding program to select for bruchid-resistant genotypes.

## Conclusions

A strong QTL for bruchid resistance on chromosome 5 was mapped in mungbean populations derived from TC1966 and V2802, suggesting the presence of the same QTL loci in both resistance sources. While physical mapping suggests the presence of another QTL on a different chromosome, co-segregation of the alleles at the QTL loci suggest strong linkage of the markers defining the QTL(s). Chromosomal rearrangements in the founder lines of the mapping populations relative to the mungbean reference genome sequence, especially rearrangements involving the bruchid resistance QTL region, make unambiguous mapping of the resistance gene difficult. Nevertheless, molecular markers predicting the resistant and susceptible genotype with up to 100 % accuracy were identified. These markers will facilitate the breeding of bruchid-resistant mungbean varieties and support the positional cloning of the resistance genes and their regulative elements.

## Methods

### Plant materials and bruchid resistance tests

A recombinant inbred line (RIL) population of TC1966 × NM92 was established as described by [12] and advanced to the F_12_ generation by single seed descent. Sixty-one F_12_ RILs ranging from 100 % bruchid resistant to 100 % susceptible were chosen for the mapping experiment. A second population was established from cross V2802 (bruchid resistant) × NM94 (bruchid-susceptible) and 150 lines were advanced, also by single seed descent, to the F_7_ generation. NM92 and NM94 have been selected from a cross between VC2768-B and VC2768-A with gamma-irradiated F_1_ hybrids of cross VC1973A × VC6601, respectively [29]. For V2802 the pedigree is unknown, and TC1966 *V. radiata* var. *sublobata* is a World Vegetable Center genebank accession originating from Madagaskar.

The plants were grown in greenhouses during the spring and autumn seasons in pots and seed was harvested at maturity. Bruchid-resistance tests were performed on 61 F_12_ families of TC1966 × NM92 and 141 families over three generations (F_3_, F_5_ and F_7_) for V2802 × NM94 in three biological replicates of 40 seeds each, using a method described in [12]. Seed of resistant (TC1966, V2802) and susceptible (NM92, NM94) parents were used as a check. Each seed batch was inoculated with 20 newly emerged bruchid adults for mating and laying eggs on the seeds. Seven days after inoculation, all adults were removed and presence of at least 2 eggs per seed was checked. After 30 days of incubation at room temperature, the proportion of damaged seed and the number of emerged bruchid beetles was determined.

### Genotyping

DNA was extracted from the cotyledon and the shoot apex of sprouts of the parental lines and from pooled plant material of 10 plants per family of 61 F_12_ families of TC1966 × NM92 and from 141 F_7_ families of V2802 × NM94 using the DNEasy Plant Mini Kit (Qiagen) according to the instructions of the supplier. DNA was quantified on a Qubit fluorometer using a Qubit assay kit (Invitrogen). GBS library preparation using restriction enzyme *ApeKI*, barcode and adapter sequences were as described in [25]. Before ligation, quality control of the fragmentation of the genomic DNA was tested by comparing digested, un-digested and mock-digested (reaction contained restriction enzyme buffer, but no enzyme) DNA with each other on 1 % agarose gels.

Size selection of the adapter-ligated restriction fragments was performed after electrophoresis at 145 V for 45 min on 6 % polyacrylamide gels in tris-borate-EDTA (TBE) buffer side-by side with a 50 bp DNA ladder as a size marker. The gel was stained with SYBR Gold diluted 10,000-fold in 0.5 × TBE buffer for 10 min. The DNA bands were visualized under ultraviolet light and the smear of DNA fragments in the size range between 300 and 500 bp was cut out from the gel. The gel pieces containing DNA of one lane were placed each in a 0.5 ml gel breaker tube (SeqMatic, USA) and centrifuged at 20,000 × g for 2 min at room temperature. 200 μl ultra-pure water was added to each tube, and the tubes were shaken for 2 h on a rotating orbital shaker at room temperature. The liquid and the gel debris were transferred to a spin column (Ambion, AM10065) and centrifuged for 5 min at top speed. The eluate was forwarded for sequencing to the High Throughput Genomics Core Facility of the Biodiversity Research Center, Academia Sinica, Taipei, Taiwan. The DNA quantity and fragment size in the libraries was verified on a bioanalyzer, subsequently the reactions were sequenced on an Illumina HiSeq 2500 apparatus. Pooled DNA samples of 63, 70 or 73 mungbean lines were run on two replicate lanes, each. The Fastq-files of the raw reads were processed in Tassel on an IBM × 3500–4 workstation. The Tassel 5 standalone pipeline was followed as outlined in the manual. Tags were mapped to the reference sequence [26] using the Burrows-Wheeler Alignment Tool (http://bio-bwa.sourceforge.net/bwa.shtml). SNPs were exported to Microsoft Excel and segregating loci that were homozygote in the parents and had less than 30 % missing data were used for genetic mapping and QTL analysis.

#### Genetic mapping and QTL analysis

Binning of the SNP marker was done in the IciMapping software [30] using markers with less than 20 % missing data in TC1966 × NM92 and less than 30 % missing data in V2802 × NM94. Genetic maps were constructed with the IciMapping software after grouping the binned markers at a logarithm of odds (LOD) of 6. QTL analysis was done with the IciMapping software using interval and inclusive composite interval mapping on genetic maps as well as on markers ordered according to their physical map position in the reference sequence of VC1973 [26]. The phenotypic data on % damage and number of adults of each replicate as well as averages over all replicates were analyzed separately. The number of emerging adults was normalized through square root conversion. Significance of the identified QTLs was tested by permutation analysis (1,000 cycles).

#### Marker validation

Selected SNP markers associated with bruchid resistance in V2802 × NM94 (F_7_) and TC1966 × NM94 (F_12_) were converted to CAPS markers using the CAPS designer tool (https://solgenomics.wur.nl/tools/caps_designer/caps_input.pl). SNPs that could not be transferred to CAPS were converted to dCAPS according to [31] using the dCAPS finder (http://helix.wustl.edu/dcaps/dcaps.html). For SNP markers on chromosomes 1, 2, 7 and 10 tetra markers were designed in primer3. In addition to SNP markers identified in the present study, markers previously found being associated with bruchid resistance including DMB SSR-158 [12] for population V2802 × NM94, and for TC1966 in addition to MB-87 [21]. OPW02a4, 34480, 34458 and 779 [13] were included as controls (Table 3). Primer sequences of markers were mapped to the reference genome using the web blast tool of the Crop Genomics Lab of the Seoul National University, Republic of Korea (http://plantgenomics.snu.ac.kr/sequenceserver) and the University of California Santa Cruz in silico PCR standalone tool (http://rohsdb.cmb.usc.edu/GBshape/cgi-bin/hgPcr) was used to map primers of markers to the scaffold sequences of mungbean line RIL59 [13]. Genomic DNA was either available from the GBS experiment, or was extracted from fresh leaf tissues according to [32]. Polymerase chain reaction (PCR) was performed in 15 μl reactions containing 0.2 μM of each primer, 200 μM of deoxyribonucleotides, 50 mM KCl, 10 mM Tris HCl (pH 8.3), 1.5 mM MgCl_2_, 25 ng of DNA and 0.5 unit of Taq DNA polymerase. The amplification profile was 94 °C for 5 min, followed by 30 cycles of 94 °C for 30 s, 55 °C for 45 s, 72 °C for 45 s, and final extension for 7 min at 72 °C. The annealing temperature was adjusted for each primer combination. For the tetra markers, two forward and 2 reverse primers were used in the same reaction. The CAPS markers were subsequently digested with restriction enzymes as listed in Table 3. PCR products or restriction fragments (3 μl) were size-fractionated on 6 % non-denaturing polyacrylamide gels in 0.5 × TBE buffer. After electrophoresis, the gels were stained with 5 μg/mL^−1^ ethidium bromide and the bands were visualized under ultraviolet light.

**Table 3:** Markers used in the study

Name	Chromosome	Position	Forward primer	Reverse primer	Enzyme	Resistant allele fragment size	Susceptible allele fragment size	Reference
dCAPS2	3	3:10,431,528	CCTCCTCTGTTGGGAAATCA	TCTGAAGGCCTGTGTTAAGCT	Hind III	307	286 21	this study
dCAPS3	4	4:15,255,162	AGTACGGCCTCAAACAGTGG	GAAAATTACAATCAAATGGAGCT	SacI	303	283 20	this study
CAPS1	5	5:5,178,332	ACTTCACTGGGTGGACTTGC	ATTCTCAGGCATGGTCAAGG	TaqI	89 364	453	this study
CAPS2	5	5: 5,179,402	AGGTGAAATTGGTTGGAAGG	CCCATGTCAGAAGCATCATC	Hpy 188III	104 129 43 14 37	104 172 14 37	this study
dCAPS1	5	5,454,538	AGCTGTGGAATGACGACTAG	TTACAACACCCAGTGCGTTC	SPE1	324	304 20	this study
CAPS3	5	5: 5,622,070	TGCTCAGCTGCTATACCAAGA	CACAATGCCTGATGGAGAGA	XcmI	46 398	444	this study
CAPS4	5	5: 5,662,479	GAACCAGTTCAAGCGACTCC	CGAACTTAGAGGCCAAAACG	BanI	108 193	301	this study
CAPS6	5	5:5,730,691	CTGAATGGGTTTATGCGTTG	ATCAATGGCCCCTCTCTTTT	PsiI	306	139 167	this study
CAPS12	5	5:5,953,917	TGCATGTCAACGAAAACTCA	GTAGAGGGGGTTTTCCGAAG	TaqI or HinfI	188 (190) 122 (120)	310	this study
CAPS13	5	5:5,974,663	CGCAGCGAATGTTATCACTG	TTGCTGTGAAATTGCAGCTC	TaqI	221 180	401	this study
CAPS8	5	5:6,992,170	CGCCCTCCGTGTATTCTAAA	GGCTGCTTCACTTACCAAGC	BstYI	419	330 89	this study
CAPS14	5	5:6,066,948	CCGAGCATTGAGGTTGGTAT	CTAAGGCGAGCTGCTGAAGT	NheI	322	165 157	this study
CAPS9	5	5:7,212,649	TGGCATGAAATGAGTTAAAAGTG	TCCTGAACTTGGGGTTATGG	RsaI	66 366	66 173 193	this study
CAPS11	5	5:7,551,254	ACAAGCTGATGGGCAAACTC	GACGGATCCGAGTGTTTGTT	AseI	100 238	100 113 125	this study
TETRA1	1	26,370,595	O:CCGAAGATGTGTGATTCATG I:GACGATACTTGTCCAGATAT	O:GAAGGGATTTTGTTAGGAGT I:AGCACTCAAGATGAAAGTGATC	-			this study
TETRA2	2	23,741,639	O:ACTATCTGACCGAAAGGAA I:TTGGTACCAAATTCTGCACT	O:ATCTGCTGACAGGAGAATTCA I:ATCTTACGGTGAAGGACATT	-			this study
TETRA3	7	13,713,780	O:TAGCTGGTCCGTGTACTTTA I:TTTCCATTGTGGGTCGTGGAGT	O:GGAACTATGCTTTGGGACTT I:ATTCTTGTAGCATCATCAAAACT	-			this study
TETRA4	10	3,159,416	O:ATACTGGAGGGTTGTTTCTA I:TAAGCGTGCGCAGCCATAAACAAG	O:CGGTCTCAGAATCATAGTCTTG I:GGGTTTTTTCGGAAATTCAAAG	-			this study
DMB-SSR-158	5	5:5,597,891 - 5:5,590,645^a^	TGGAAAATTTGCAGCAGTTG	ATTGATGGAGGGCGGAAGTA		300-400		[11, 12]
779	5	7351532-7351612	CTAATAAATCATCTATACgTCTCTC	ATTgCTATTTAgCgAATAATAgTAC				[13]
OPW 02a4	5	6743545 – 6745026^a^	CCAAAggAgTCgAgTgAAACT	CAACAACCCTTCCTCTATCTC		400 – 1,100		[13]
Vr34480	?	?	AATTCTTgATTggTCCACATg	AAAAAATTACACCTCgTTCg		500		[13]
MB-87	?	Fwrd:3:11,421,121	TCCCTTGTGGGAGATCCT	CTTTGCCACACTCCTTGC		~300		[21]
		4:15,255,162						
		5:5,602,099						
		Rev: −^b^						

O: outer primer, I: inner primer for tetra primer, ^a^) the predicted fragment length according to the reference sequence is larger than the fragment obtained in both populations. ^b^) forward and reverse primer do not match to a common scaffold or chromosome of the reference sequence

## Abbreviations

AFLP, amplified fragment length polymorphism; CAPS, cleaved amplified polymorphic sequences; cM, centimorgan; dCAPS, derived cleaved amplified polymorphic sequences; GBS, genotyping-by-sequencing; I, inner primer; LOD, logarithm of odds; O, outer primer; PCR, polymerase chain reaction; QTL, quantitative trait locus; RIL, recombinant inbred line; SNP, single nucleotide polymorphism; sp., species; TBE, tris-borate-EDTA; var., variety

## Additional files

Additional file 1: Table S1. List of all SNPs that are homozygous in the mapping parents and map to chromosome 1–11 of the mungbean reference sequence (Kang YJ, Kim SK, Kim MY, Lestari P, Kim KH, Ha BK, et al. Genome sequence of mungbean and insights into evolution within Vigna species. Nat Commun. 2014; doi:10.1038/ncomms6443). (XLSX 343 kb) Additional file 2: Table S2. Interval mapping of bruchid resistance on physical maps of populations TC1966 x NM94 and V2802. (DOCX 14 kb) Additional file 3: Table S3. Marker genotypes of families of the mapping populations TC1966 × NM92 and V2802 × NM94 at bruchid resistance loci. (XLSX 18 kb) Additional file 4: Table S4. Gene content of the reference genome VC1973 in the chromosome 5 QTL interval. (DOCX 18 kb) Additional file 5: Figure S1. Genotypes of markers for QTLs detected by interval mapping on chromosomes 1, 2, 7 and 10 of TC1966 × NM92 or V2802 × NM94. (DOCX 1189 kb)
