# Supplementary material for: Identification of single nucleotide polymorphism markers associated with resistance to bruchids (Callosobruchus spp.) in wild mungbean (Vigna radiata var. sublobata) and cultivated V. radiata through genotyping by sequencing and quantitative trait locus analysis
Source: BMC Plant Biol. 2016 Jul 15;16:159. doi: 10.1186/s12870-016-0847-8 (PMC4946214; doi:10.1186/s12870-016-0847-8)
Supplement: Additional file 2: Table S2. — Interval mapping of bruchid resistance on physical maps of populations TC1966 x NM94 and V2802. (DOCX 14 kb) [file 12870_2016_847_MOESM2_ESM.docx]

Supplemental Table 2: Interval mapping of bruchid resistance on physical maps of populations TC1966 x NM94 and V2802. Significant QTLs for reduced seed damage are located on chromosome 1, 3, 4, 5 and 10 in TC1966 x NM92 and on chromosomes 2, 3, 4, 5 and 10 in V2802 x NM94.

| **Population** | **Chr.** | **Left marker** | **Right marker** | **LOD** |
| --- | --- | --- | --- | --- |
| TC1966 x NM92 | 1 | 1:26,370,595 | 1:28,364,419 | 31.9 |
| V2802 x NM94 | 2 | 2:23,741,639 | 2:24,208,814 | 16.4 |
| TC1966 x NM92, V2802 x NM94 | 3 | 3:10,257,752 | 3:11,651,817 | 40.2, 28.1 |
|  | 4 | 4: 15,495,366 | 4:15,684,641 | 43.3, 40.4 |
|  | 5 | 5:5,179,402 | 15,541,296 | 39.9, 41.2 |
| TC1966 x NM92 | 7 | 7:13,713,780 | 7:14,594,366 | 10.0 |
| TC1966 x NM92  V2802 x NM94 | 10 | 10:2,772,296 | 10: 3,814,194 | 12.2, 12.4 |
