# Supplementary material for: Identification of single nucleotide polymorphism markers associated with resistance to bruchids (Callosobruchus spp.) in wild mungbean (Vigna radiata var. sublobata) and cultivated V. radiata through genotyping by sequencing and quantitative trait locus analysis
Source: BMC Plant Biol. 2016 Jul 15;16:159. doi: 10.1186/s12870-016-0847-8 (PMC4946214; doi:10.1186/s12870-016-0847-8)
Supplement: Additional file 4: Table S4. — Gene content of the reference genome VC1973 in the chromosome 5 QTL interval. (DOCX 18 kb) [file 12870_2016_847_MOESM4_ESM.docx]

Supplemental Table 4. Gene content of the reference genome VC1973 in the chromosome 5 QTL interval between markers located at positions 5,178,332 and 6,066,948

| Region | Gene name | Function | SNP in gene sequence in TC1966 | SNP in gene sequence in V2802 | Predicted amino acid change |
| --- | --- | --- | --- | --- | --- |
| Vr05:5175003..5180305 | Vradi05g03780.1 | uncharacterized mRNA |  | 5,176,111 A/T  5,176,570 C/T  5,177,371 G/t  5,177,413 A/c | -  -  -  - |
|  |  |  | 5,177,704 A/G  5,178,332 A/G  5,178,375 G/T  5,178,940 G/T  5,179,150 C/T  5,179,235 G/A  5,179,386 C/T  5,179,402 C/T | | -  R: D, S: N  R: V, S: G  -  R: R, S: M  -  R: F, S: S  - |
| Vr05:5182693..5186918 | Vradi05g03790.1 | rac-like GTP-binding protein ARAC8 | - | - |  |
| Vr05:5202076..5204576 | Vradi05g03800.1 | probable inactive receptor kinase At2g26730 | 5,204,049 C/M  5,204,076 A/M  5,204,100 A/W |  | - |
| Vr05:5236102..523868 | Vradi05g03810.1 | probable polygalacturonase non-catalytic subunit At1g60390 isoform X1 | - | - |  |
| Vr05:5278571..5280016 | Vradi05g03820.1 | hypothetical protein VIGAN_11031400 | - | - |  |
| Vr05:5284760..5286543 | Vradi05g03830.1 | hypothetical protein LR48_Vigan04g051700 | - | - |  |
| Vr05:5300271..5304169 | Vradi05g03840.1 | BURP domain-containing protein 9-like | - | - |  |
| Vr05:5338888..5339265 | Vradi05g03850.1 | dehydration-responsive protein RD22-like | - | - |  |
| Vr05:5367245..5367592 | Vradi05g03860.1 | dehydration-responsive protein RD22-like [ | - | - |  |
| Vr05:5389200..5390826 | Vradi05g03870.1 | BURP domain-containing protein 9-like | - | - |  |
| 5620723..5623987 | Vradi05g03980.1 | probable alpha,alpha-trehalose-phosphate synthase [UDP-forming] 9 (LOC106762884), mRNA | 5,622,070 A/G | | R: P, S: S |
|  |  |  | - | 5,622,083 G/T | - |
| Vr05:5634795..5635507 | Vradi05g03990.1 | ethylene-responsive transcription factor AIL6 (LOC106755157), transcript variant X2, mRNA | - | - | - |
| Vr05:5646107..5649419 | Vradi05g04000.1 | spermidine synthase 1-like (LOC106760240), mRNA | - | - | - |
| Vr05:5659444..5662794 | Vradi05g04010.1 | cyclic nucleotide-gated ion channel 16 (LOC106760241), mRNA | 5,662,479 A/G  5,662,485 G/C | | -  - |
| Vr05:5674666..5680262 | Vradi05g04020.1 | probable 26S proteasome complex subunit sem1-2 (LOC106761081), mRNA | - | - | - |
| Vr05:5693618..5694545 | Vradi05g04030.1 | uncharacterized LOC106761581 (LOC106761581), mRNA | - | - | - |
| Vr05:5728779..5730593 | Vradi05g04040.1 | probable polygalacturonase non-catalytic subunit At1g60390 (LOC106762767) | - | - | - |
| Vr05:5802317..5812432 | Vradi05g04050.1 | EXECUTER 2, chloroplastic (LOC106762252), mRNA |  | 5,805,624 C/T  5,805,930 G/A |  |
|  |  |  | 5,808,664 A/G | | - |
| Vr05:5844288..5849927 | Vradi05g04060.1 | alpha-L-arabinofuranosidase 1-like (LOC106753719), mRNA | - | - | - |
| Vr05:5854176..5859875 | Vradi05g04070.1 | serine/threonine-protein kinase HT1-like (LOC106762690), mRNA | - | - | - |
| Vr05:5872454..5875665 | Vradi05g04080 | uncharacterized LOC106762547 (LOC106762547), transcript variant X2, mRNA | - | - | - |
| Vr05:5876388..5881205 | Vradi05g04090.1 | carbonic anhydrase 2-like | 5,877,013 A/G  5,877,096 T/A | | - |
| Vr05:5896285..5903903 | Vradi05g04100.2 | peroxisomal membrane protein PEX14-like | 5,898,074 A/G  5,898,090 T/A |  | - |
|  |  |  |  | 5,898,117 G/A | - |
| Vr05:5924837..5925951 | Vradi05g04110.1 | uncharacterized LOC106761171 (LOC106761171), mRNA | - | - | - |
| Vr05:5939333..5941344 | Vradi05g04120.1 | probable inactive poly [ADP-ribose] polymerase SRO2 | - | - | - |
| Vr05:5971494..6038339 | Vradi05g04130.1 | probable LRR receptor-like serine/threonine-protein kinase At4g36180 |  | 5,974,663 C/T |  |
|  |  |  | 5,974,765 T/A | | R: N, S: K |
|  |  |  |  | 5,979,412 T/G  6,011,645 A/G | R: C, S: K |
| Vr05:6056562..6057886 | Vradi05g04140.1 | hypothetical protein LR48_Vigan04g060600 | - | - | - |
| Vr05:6060050..6081357 | Vradi05g04150.1 | PREDICTED: pantothenate kinase 2-like | 6,066,948 T/A | |  |
|  |  |  |  |  |  |
