# Supplementary material for: Identification of single nucleotide polymorphism markers associated with resistance to bruchids (Callosobruchus spp.) in wild mungbean (Vigna radiata var. sublobata) and cultivated V. radiata through genotyping by sequencing and quantitative trait locus analysis
Source: BMC Plant Biol. 2016 Jul 15;16:159. doi: 10.1186/s12870-016-0847-8 (PMC4946214; doi:10.1186/s12870-016-0847-8)
Supplement: Additional file 5: Figure S1. — Genotypes of markers for QTLs detected by interval mapping on chromosomes 1, 2, 7 and 10 of TC1966 × NM92 or V2802 × NM94. (DOCX 1189 kb) [file 12870_2016_847_MOESM5_ESM.docx]

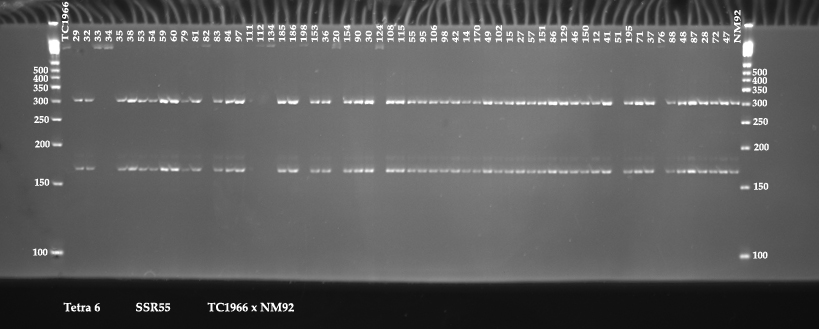
a)

Seed damage (%)

0.0

0.0

0.0

0.0

0.0

0.0

0.0

0.0

0.0

0.0

0.0

0.0

0.0

0.0

0.0

0.0

0.0

0.0

0.0

0.0

0.0

0.0

7.5

17.5

20.0

17.5

37.5

45.0

95.0

100.0

97.5

100.0

92.5

97.5

100.0

100.0

100.0

97.5

100.0

100.0

100.0

100.0

97.5

97.5

100.0

100.0

100.0

100.0

100.0

100.0

100.0

100.0

100.0

87.5

100.0

100.0

100.0

97.5

100.0

100.0

100.0

0.0

0.0

0.0

0.0

0.0

0.0

0.0

0.0

0.0

0.0

0.0

0.0

0.0

0.0

0.0

0.0

0.0

0.0

0.0

0.0

0.0

0.0

0.0

0.0

0.0

0.0

0.0

0.0

0.0

0.0

0.0

0.0

0.0

0.0

0.0

0.0

0.0

0.0

0.0

0.0

0.0

0.0

0.0

0.0

0.0

0.0

0.0

0.0

0.0

0.0

0.0

2.7

4.7

6.7

7.3

11.3

12.0

12.0

16.0

17.3

18.7

19.3

20.7

22.3

28.0

29.3

29.3

30.0

30.0

31.3

0.0

0.0

0.0

0.0

0.0

0.0

0.0

0.0

0.0

0.0

0.0

0.0

0.0

0.0

0.0

0.0

0.0

0.0

0.0

0.0

0.0

0.0

7.5

17.5

20.0

17.5

37.5

45.0

95.0

100.0

97.5

100.0

92.5

97.5

100.0

100.0

100.0

97.5

100.0

100.0

100.0

100.0

97.5

97.5

100.0

100.0

100.0

100.0

100.0

100.0

100.0

100.0

100.0

87.5

100.0

100.0

100.0

97.5

100.0

100.0

100.0


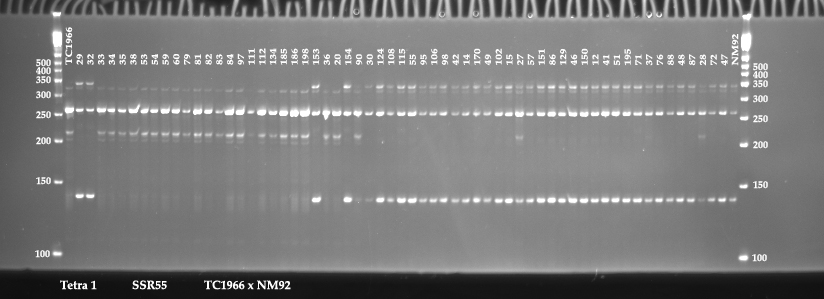


0.0

0.0

0.0

0.0

0.0

0.0

0.0

0.0

0.0

0.0

0.0

0.0

0.0

0.0

0.0

0.0

0.0

0.0

0.0

0.0

0.0

0.0

7.5

17.5

20.0

17.5

37.5

45.0

95.0

100.0

97.5

100.0

92.5

97.5

100.0

100.0

100.0

97.5

100.0

100.0

100.0

100.0

97.5

97.5

100.0

100.0

100.0

100.0

100.0

100.0

100.0

100.0

100.0

87.5

100.0

100.0

100.0

97.5

100.0

100.0

100.0

b)

Seed damage (%)

0.0

0.0

0.0

0.0

0.0

0.0

0.0

0.0

0.0

0.0

0.0

0.0

0.0

0.0

0.0

0.0

0.0

0.0

0.0

0.0

0.0

0.0

7.5

17.5

20.0

17.5

37.5

45.0

95.0

100.0

97.5

100.0

92.5

97.5

100.0

100.0

100.0

97.5

100.0

100.0

100.0

100.0

97.5

97.5

100.0

100.0

100.0

100.0

100.0

100.0

100.0

100.0

100.0

87.5

100.0

100.0

100.0

97.5

100.0

100.0

100.0


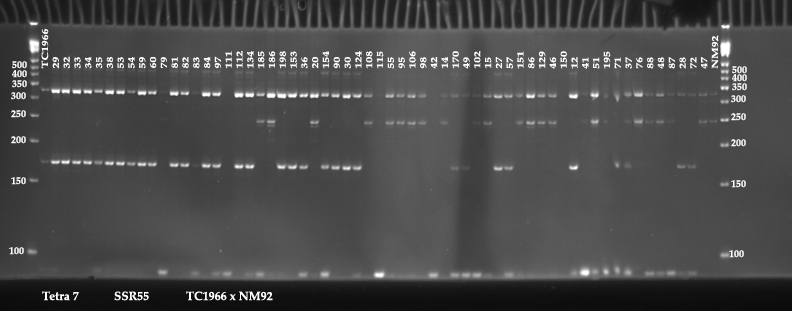
c)

Seed damage (%)


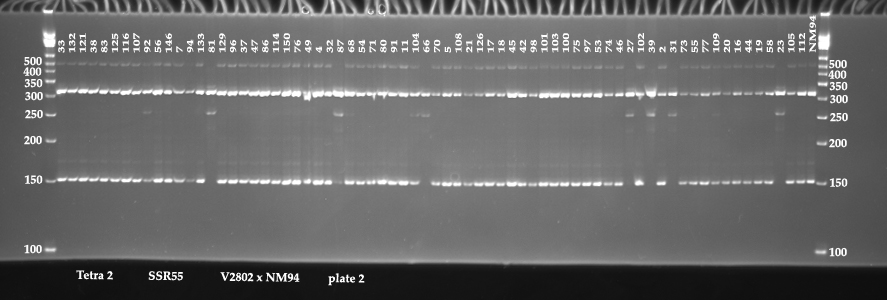

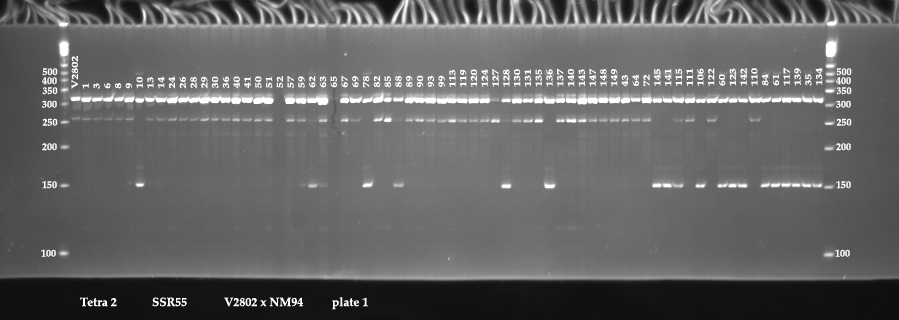
d)

2.7 – 33.3% seed damage resistant

32-100% damaged seed

100% resistant


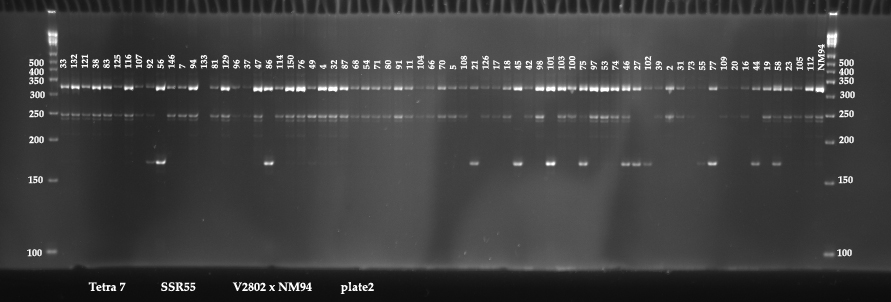

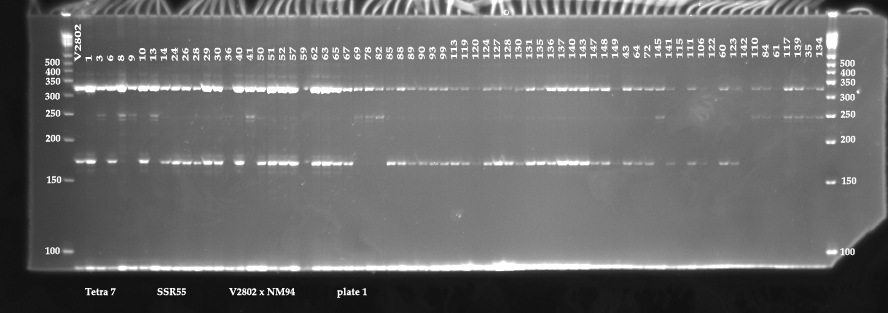


2.7 – 33.3% seed damage resistant

100% resistant

e)

32-100% damaged seed

Supplemental Figure 1. Marker genotypes in TC1966 x NM92 (a-c) and V2802 x NM94 (d, e) for QTLs detected by interval mapping. a) Tetra marker 1 (chromosome 1, position 26,370,595 bp), b) tetra marker 3 (chromosome 7, position 13,713,780 bp), c) tetra marker 4 (chromosome 10, 3,159,416 bp), d) tetra marker 2 (chromosome 2, position 23,741,639 bp), e) tetra marker 4 (chromosome 10, position 3,159,416).
